# Non-invasive three-dimension control of light between turbid layers using a surface quasi-point light source for precorrection

**DOI:** 10.1038/s41598-017-10450-7

**Published:** 2017-08-29

**Authors:** Mu Qiao, Honglin Liu, Guanghui Pang, Shensheng Han

**Affiliations:** 1 Key Laboratory for Quantum Optics and Center for Cold Atom Physics, Shanghai Institute of Optics and Fine Mechanics, Chinese Academy of Sciences, Shanghai, 201800 China; 2 University of Chinese Academy of Sciences, Beijing, 100049 China

## Abstract

Manipulating light non-invasively through inhomogeneous media is an attractive goal in many disciplines. Wavefront shaping and optical phase conjugation can focus light to a point. Transmission matrix method can control light on multiple output modes simultaneously. Here we report a non-invasive approach which enables three-dimension (3D) light control between two turbid layers. A digital optical phase conjugation mirror measured and conjugated the diffused wavefront, which originated from a quasi-point source on the front turbid layer and passed through the back turbid layer. And then, because of memory effect, the phase-conjugated wavefront could be used as a carrier wave to transport a pre-calculated wavefront through the back turbid layer. The pre-calculated wavefront could project a desired 3D light field inside the sample, which, in our experiments, consisted of two 220-grid ground glass plates spaced by a 20 mm distance. The controllable range of light, according to the memory effect, was calculated to be 80 mrad in solid angle and 16 mm on z-axis. Due to the 3D light control ability, our approach may find applications in photodynamic therapy and optogenetics. Besides, our approach can also be combined with ghost imaging or compressed sensing to achieve 3D imaging between turbid layers.

## Introduction

Control of light behind heterogeneous media is a fundamental goal in many fields ranging from photodynamic therapy^1–3^ and optogenetics^4–6^ to atmospheric optics^7–9^ and biomedical optics^10–13^. Considerable efforts have been made with methods such as adaptive optics technologies^14–16^, wavefront shaping^17–22^, transmission matrix^23–29^, optical phase conjugation^30–37^, and memory effect^38–40^. Guidestar-assisted methods^19^, using either optical phase conjugation or wavefront shaping to suppress turbidity, can focus light through or into scattering media. Transmission matrix method measures the matrix elements connecting the input and output modes of a scattering layer, and then modifies the input modes to accumulate more energy to the intended modes on the output plane.

Memory effect^39,40^, stating as that tilting of the incident light beam within a certain angular range results in shifted, but highly correlated speckle patterns, is an effective tool for applications dealing with thin scattering layers. Via memory effect, scanning of a light focus behind a turbid layer can be done on x-y plane^41,42^ or along z-direction^43^ by digitally superposing a linear or quadratic phase pattern on the optimized one displayed on spatial light modulator (SLM). Moreover, several memory effect based imaging techniques^44–46^ were demonstrated, which had characteristics such as incoherent illumination, non-invasion and optical speckle-scale resolution.

**Figure 1:**
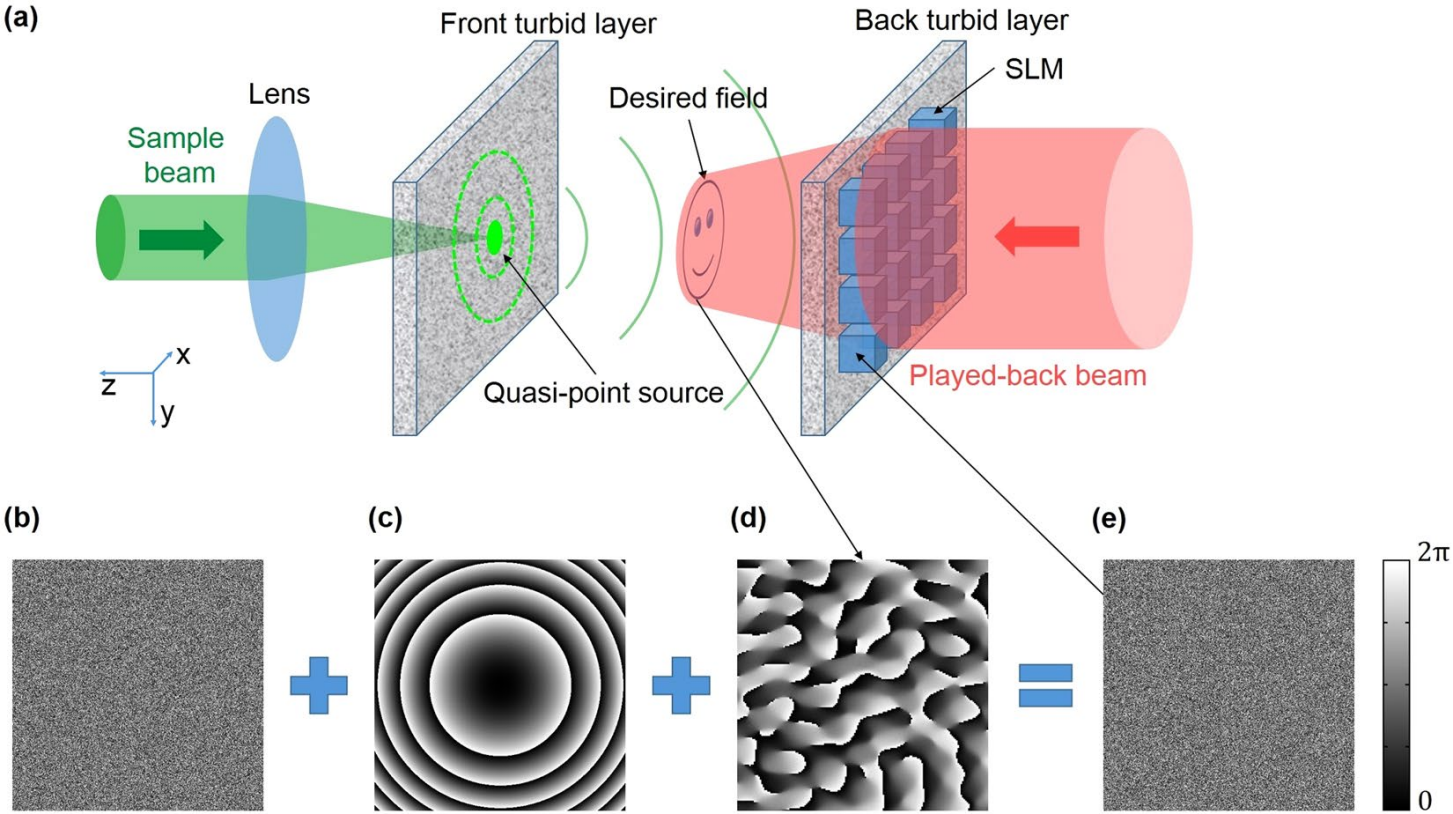
The Schematic of the presented approach for light control between two turbid layers. (**a**) The schematic of our experimental setup. The scenario we consider here is a light-transparent space confined by two turbid layers. The sample beam (green path) is focused by a lens to form a quasi-point source on the internal surface (right surface) of the front turbid layer (left one), which is used as a reference spot for pre-characterization of the back turbid layer (right one). A spatial light modulator is imaged onto the external surface (right surface) of the back turbid layer (imaging lens not shown here, see Fig. S1 in Supplementary Information). The played-back reference beam (red path) is modulated by the SLM according to the applied phase map, which is a stacked one consisting of three parts shown in (**b**), (**c**) and (**d**), respectively. (**b**) The conjugated phase map of the sample beam. (**c**) A quadratic phase map which is used to cancel out the quasi-spherical wave emitted from the quasi-point source. (**d**) A pre-calculated phase map for the intended intensity pattern (presented by the smiling face). The modulated reference beam can be seen as a loaded carrier wave with the phase-conjugated wave and the quadratic wave together as the carrier, and the pre-calculated wavefront as the load. The carrier wave suppresses the turbidity of the back turbid layer and thus can transport the pre-calculated wavefront through this layer, which would generated/projected the intended intensity pattern.

Here, we demonstrated an approach combining memory effect and optical phase conjugation technique for 3D light control between two turbid layers (in our case, two 220-grit ground glass plates spaced by a 20 mm distance) in a completely non-invasive way with diffraction-limited resolution. We used a lens to focus the sample beam onto the external surface of the front turbid layer (left surface of the left layer, Fig. 1a), resulting in a micrometer-scale quasi-point source on the internal surface of the front layer (right surface, Fig. 1a), which emitted a quasi-spherical wave toward the back turbid layer (right layer, Fig. 1a), where the wavefont was strongly diffused. Then a digital optical phase conjugation^34^ (DOPC) system was used to measure and conjugate the diffused wavefront. By taking advantage of the memory effect, we used the phase-conjugated wavefront as a carrier wave to transport a pre-calculated wavefront through the back turbid layer. Then, the pre-calculated wavefront could project a desired 3D field, just like 3D holographic projection, at intended positions in between the two turbid layers. A direct application of our approach is to focus light into an optically transparent medium confined by turbid layers, such as crustaceans and eggs, without assistant of any real^47,48^ or virtual^32,34,35^ guidestars inside the medium, so our approach is completely non-invasive.

## Results

### Principle

For thin scattering layers, the memory effect states that a tilt of the incident light field results in an equal amount of tilt of the output field. We know that a tilt of light field corresponds to a shift in wavevector space, which can be equally described in coordinate space as multiplying the initial field by a plane wave. So the memory effect can be expressed as

1 $$T(\exp (i{k}_{x}x)\ast A(x))=\exp (i{k}_{x}x)\ast T(A(x))$$

where *T* represents the transmission function characterizing a scattering layer^27,29^, *k*_*x*_ represents the wavevector, *x* represents the coordinate, and $\exp (i{k}_{x}x)$ represents the tilt of an arbitrary incident light field $A(x)$. Here we further exploit the memory effect to gain more control over light behind thin scattering layers rather than just shifting an interference based focus on the x-y plane or along the z-direction.

Figure 1a presents the schematic of our approach. A lens with short focal length focuses a sample beam onto the external surface (the left side) of the front ground glass plate (the left one), resulting in a micrometer-scale focus which, serving as a quasi-point light source, launches a quasi-spherical wave toward the back ground glass plate (the right one). For clarity and simplicity, we will call the back ground glass plate as the modulating layer below. The field ${E}_{out}$ on the external surface (the right side) of the modulating layer can be read as

2 $${E}_{out}=T{E}_{sphe}$$

where ${E}_{sphe}$ denotes the quasi-spherical wave on the internal surface (left side) of the modulating layer and *T* represents the transmission function of the modulating layer. The outside surface (right side) of the modulating layer is imaged onto a phase-only spatial light modulator by a commercial lens (not shown in Fig. 1a, see Supplementary Information Fig. S1) - which eliminates the convolution calculation involved by propagation of light between these two planes, and thus enables direct phase control of the field on the external surface of the modulating layer by loading a phase pattern to the SLM. As the first step of our approach (recording step), interference patterns created by ${E}_{out}$ and a reference beam are recorded with the DOPC system using a 4-phase stepping method^49^. In the second step (playback step), the sample beam is switched off and the SLM displays a ‘stacked’ phase pattern which is a superposition of three patterns: a conjugated phase pattern ${E}_{conj}$ calculated from the interference patterns recorded in the first step, a quadratic phase pattern ${E}_{quad}$ used to cancel out the quasi-spherical wave ${E}_{sphe}$, and a phase pattern *H* which is calculated from an intended 3D intensity distribution using an iterative Fourier transformation algorithm^50^. The reference beam is modulated by the SLM and travels back to the sample. The field on the external surface of the modulating layer can be written as

3 $$E{^{\prime} }_{out}={E}_{conj}\ast {E}_{quad}\ast H$$

Substituting ${E}_{conj}={T}^{\ast }{{E}^{\ast }}_{sphe}$ (superscript * denotes the complex conjugation operation of a matrix) into equation (3), we get

4 $$E{^{\prime} }_{out}=({T}^{\ast }{{E}^{\ast }}_{sphe})\ast {E}_{quad}\ast H$$

When this field passes through the modulating layer and reaches the internal surface of this layer, the field becomes

5 $$E{^{\prime} }_{in}={T}^{T}(({T}^{\ast }{{E}^{\ast }}_{sphe})\ast {E}_{quad}\ast H)$$

where superscript *T* denotes the transposition operation of a matrix, and ${T}^{T}$^31^ denotes the time-reversed transmission function of the modulating layer and satisfies

6 $${T}^{T}{T}^{\ast }A=A$$

for arbitrary field *A*. The term ${E}_{quad}\ast H$ in the right side of equation (5) can be decomposed into a set of plane waves

7 $${E}_{quad}\ast H={\sum }_{l}{a}_{l}\ast \exp (i{k}_{l}x)$$

where ${a}_{l}$ is the coefficient of the $l$ th plane wave with wavevector ${k}_{l}$. Substituting equations (1), (6) and (7) into equation (5), and considering the linearity of the time-reversed transmission function ${T}^{T}$, we have

8 $$E{^{\prime} }_{in}={E}_{sphe}^{\ast }\ast {E}_{quad}\ast H$$

If the quasi-point source is infinitely small, we have ${E}_{sphe}=\exp (\frac{-ik}{2d}({x}^{2}+{y}^{2}))$ under paraxial approximation, where $k$ represents the wavevector of light, $d$ represents the distance between the two turbid layers, $x$ and $y$ represent the transverse coordinates. Then we can make ${E}_{quad}$ equal $\exp (\frac{-ik}{2d}({x}^{2}+{y}^{2}))$ to get ${E}_{sphe}^{\ast }\ast {E}_{quad}=1$ and equation (9) becomes

9 $$E{^{\prime} }_{in}=H$$

Here, we can see that the conjugated wave, serving as a carrier wave, is able to ‘escort’ *H* through the modulating layer safely so that the field $E{^{\prime} }_{in}$ on the internal surface of the modulating layer contains no information about the turbid layers. The pre-calculated wavefront *H* could project a desired 3D light field in between the two turbid layers.

It should be note that some approximations and simplifications are applied in the above derivations. First, only phase distribution is considered due to the phase-only feature of the SLM we used. Second, Equation (6) strictly holds only with complete phase conjugation, *i.e*., full control of amplitude, phase, and polarization of the diffused light over the full $4\pi$ solid angle, which is infeasible in conventional experiments. Third, the object-image transformation between the external surface of the modulating layer and the SLM plane, and the quadratic phase factor introduced by the imaging lens are not considered in the above derivations, which, however, has trivial effect on the final result. Fourth, for equation (7), only those plane waves whose directions are within the angular range where memory effect holds can be transferred through the modulating layer, and the information implicated in the remaining compositions is missing. Fifth, the derivation from equation (8) to equation (9) is based on the paraxial approximation and the assumption that the quasi-point source is infinitely small. However, in fact, a real quasi-point source has a certain size, so the quasi-spherical wave ${E}_{sphe}$ can not be exactly expressed by a quadratic phase function even under the paraxial approximation, and thus is unable to be fully compensated by another quadratic phase pattern ${E}_{quad}$. So, equation (9) should be modified to $E{^{\prime} }_{in}\approx H$.

### Simulation

From the principle above, we know that our approach needs a point source on the internal surface of the front turbid layer for pre-correction of the back turbid layer. If the wavefront ${E}_{sphe}$ emitted from the point source is a perfect spherical wave, then we can compensate it by a quadratic phase pattern ${E}_{quad}$ through the SLM. However, the scattering of the front turbid layer causes phase nonuniformity inside the quasi-point light source, resulting in a distorted quasi-spherical wave which can not be fully compensated by a quadratic phase pattern ${E}_{quad}$.

**Figure 2:**
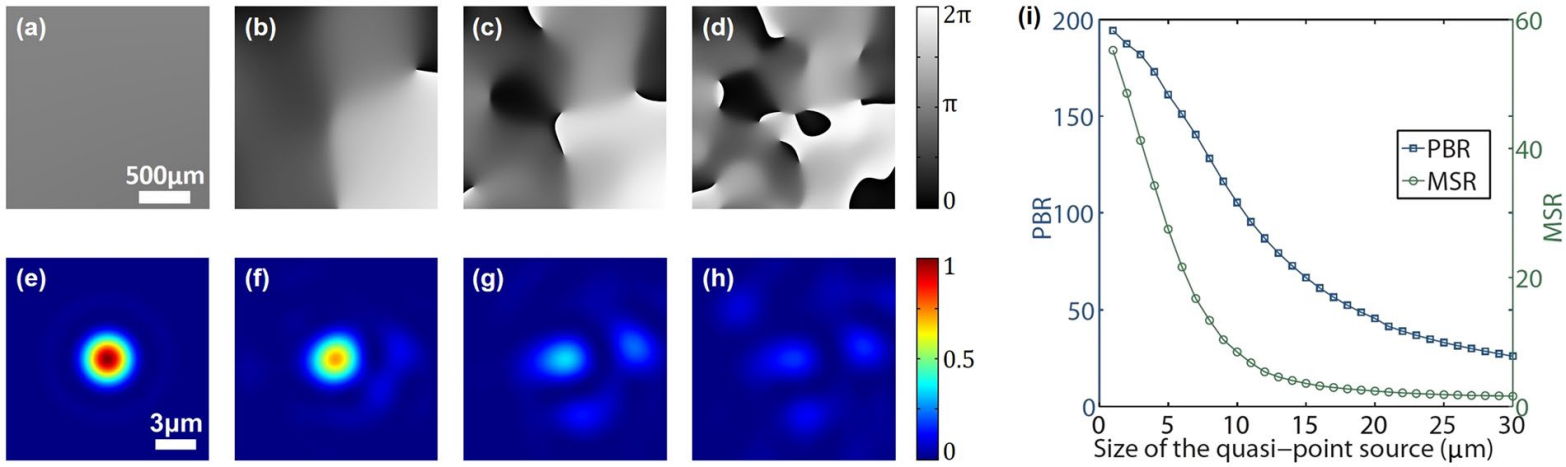
Simulation of the influences of the quasi-point source size on the performance of our approach. (**a–d**) Discrepancies between an ideal spherical wavefront and the quasi-spherical wavefronts emitted from quasi-point sources with size of 1 μm, 10 μm, 20 μm and 30 μm, respectively. The field of view is a 2 mm * 2 mm area. (**e–h**) The foci generated by superposing a lens with a focal length of 10 mm on the under-compensated wavefronts shown in a-d, respectively. The field of view is a 15 μm * 15 μm area on the focal plane. (**i**) Peak-to-background ratio (PBR, the blue curve) and maximum-to-submaximum ratio (MSR, the green curve) of the generated focus versus the size of the quasi-point source. The smooth curve is obtained by averaging 100 curves for different random phase distributions inside the quasi-point source.

In order to estimate the influences of this distortion on the performances of our approach, we carried out a simulation, in which the quasi-point light source on the internal surface of the front turbid layer was assumed to be a speckle field with diffraction-limited speckle size (λ/2, λ = 532 nm), and had a Gaussian-profile intensity distribution. The distance between the two turbid layers was set to be 20 mm. The full width of half maximum (FWHM) of the quasi-point source was set to be 1 μm to 30 μm at 1 μm step size. At each step, the distorted quasi-spherical wavefront ${E}_{sphe}$ emitted from the quasi-point source was compensated by the phase-conjugated wavefront of an ideal spherical wavefront, which had a format of $\exp (\frac{-ik}{2d}({x}^{2}+{y}^{2}))$ under paraxial approximation. The under-compensated wavefronts (discrepancies between ${E}_{sphe}$ and the ideal spherical wavefront) for the quasi-point source with sizes of 1 μm, 10 μm, 20 μm and 30 μm are shown in Fig. 2a–d, respectively. We then supposed the quadratic phase function of a lens with a focal length of 10 mm on the under-compensated wavefront at each step, and investigated the intensity distributions on the focal plane. The results for the quasi-point source with sizes of 1 μm, 10 μm, 20 μm and 30 μm are shown in Fig. 2e–h, respectively. Obviously, as the size of the quasi-point source increasing, the quality of the focus decays. More energy is transferred from the focus to the surrounding speckles, decreasing the contrast of the focus against the background. To quantify the contrast of the focus, the peak-to-background ratio (PBR), defined as the ratio of the maximum intensity of the focus to the averaged intensity of the background (a 30 μm * 30 μm area around the focus in our quantification), and the maximum-to-submaximum ratio (MSR), defined as the ratio of the maximum intensity of the focus to the maximum intensity of the sub-brightest speckle around the focus, were calculated (see Fig. 2i). These two indices describe the contrast of the focus from two different perspectives. Generally speaking, the size of the quasi-point source should be less than 10 μm for good performance. However, longer distance between the two turbid layers will have more tolerance for larger quasi-point source.

### Focusing light between two turbid layers

The sample used in our experiments consisted of two ground glass plates (DG10–220, Thorlabs, USA) separated by a 20 mm-thick air layer. The sample beam, a Gaussian beam with a diameter of 2.2 mm, was focused onto the external surface of the front ground glass plate by an aspherical lens with a focal length of 4 mm. The size of the focus was calculated to be 1.2 μm. And due to the thin thickness 1.6 μm^51^ of the ground glass plate, the size of the quasi-point source on the internal surface of this ground glass plate was no more than 10 μm, even though strong scattering was suffered when the focus passed through this layer. A commercial lens (Micro-Nikkor 105mm f/2.8, Nikon, Japan), providing 3.4X magnification, was used to image the external surface of the back ground glass plate onto the SLM (LETO, Holoeye, Germany) plane. We selected a circular area with a diameter of 6.9 mm on the center of the SLM plane as the controlled area, and its image on the sample surface had a diameter of 2 mm.

**Figure 3:**
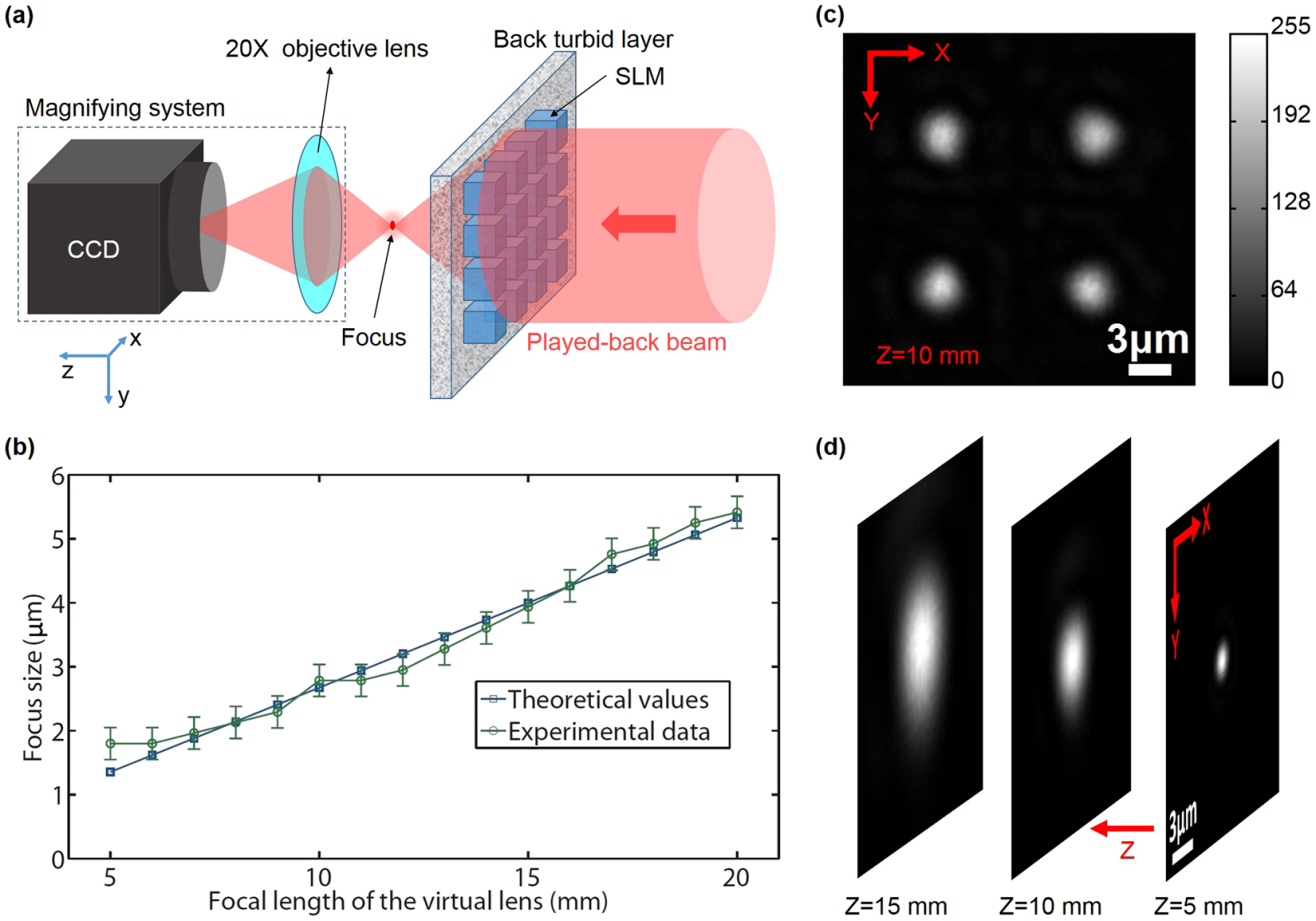
Focusing light between two ground glass plates. (**a**) Experimental setup for measurement of the focus size. A magnifying system, consisting of a CCD camera and a 20X objective lens, provides 21X magnification for precise measurement of the focus size. (**b**) The theoretical values (the blue dots) and experimental values (the green dots) of the focus size versus the distances between the focal plane and the back ground glass plate. (**c**) Observed multi-foci on an x-y plane 10 mm away from the back ground glass plate. (**d**) Observed multi-foci on z-axis. The z-positions for the three foci are 5 mm, 10 mm and 15 mm, respectively.

To focus light into our sample, the pre-calculated phase pattern $H$ should be a lens-like (quadratic) phase map, and the distance between the focus and the back ground glass plate equaled to the focal length of the virtual lens $H$. We used a CCD camera to directly measure the size of the focus. In order to reduce the measuring error caused by the finite pixel size (3.45 μm) of the CCD, a 20X objective lens (NA: 0.25; SLMPLN20x, Olympus, USA) providing 21X magnification, was used to image the focus onto the CCD’s sensor (Fig. 3a). Because of the limited space, in this measurement the front ground glass plate was removed from the experimental setup to make room for the CCD camera and the objective lens after the recording step. However, this operation did not violate the non-invasive feature of our approach, because this was only for observation and measurement of the focus, and was not necessary for the realization of light focusing. By continuously changing the focal length of the virtual lens $H$, the focus was scanned along the z-axis from z = 5 mm to z = 20 mm (the origin was at the back ground glass plate). The FWHM of the focus at each z position were measured. The results are shown in Fig. 3b (green line), which agree well with the theoretical diffraction-limited values (blue line).

To demonstrate the ability of our method to focus light simultaneously to multiple spots, we changed the $H$ to a compound lens function which was calculated by the following process. First, we calculated the lens-like function for each focus. A lens-like function has the format of $\exp (\frac{ik}{2f}({x}^{2}+{y}^{2})+i{k}_{x}x+i{k}_{y}y)$, where $f$ represents the focal length and thus determines the z position of the focus, *k*_*x*_ and *k*_*y*_ represent the directions of the wavevector and thus determine the focus position on the x-y plane. Second, we added these phase-only complex functions together to get a function $H^{\prime}$, which had both phase and amplitude distributions. Third, the phase distribution was selected from $H^{\prime}$ as the *H*. It should be noted that the second step of the above process was based on the linearity of light propagation through scattering media. The multi-foci patterns were observed by the magnification system shown in Fig. 3a. Figure 3c shows the image of four foci on an x-y plane. Figure 3d shows the images of three foci at different z positions. The size of each focus in the multi-foci focusing case is still diffraction-limited.

Due to the loss of amplitude information in *H*, there are some distortions between the intended and the observed patterns. In the multi-foci case, the distortions appear as high-order foci surrounding the intended foci, which are caused by the high-order harmonics in *H* (See Supplementary Information Fig. S2).

### Controlling light between two turbid layers

To extend our approach to more than just focus light between two turbid layers, we wish to generate more complex intensity distributions/patterns (such as letters) in target positions between two turbid layers.

**Figure 4:**
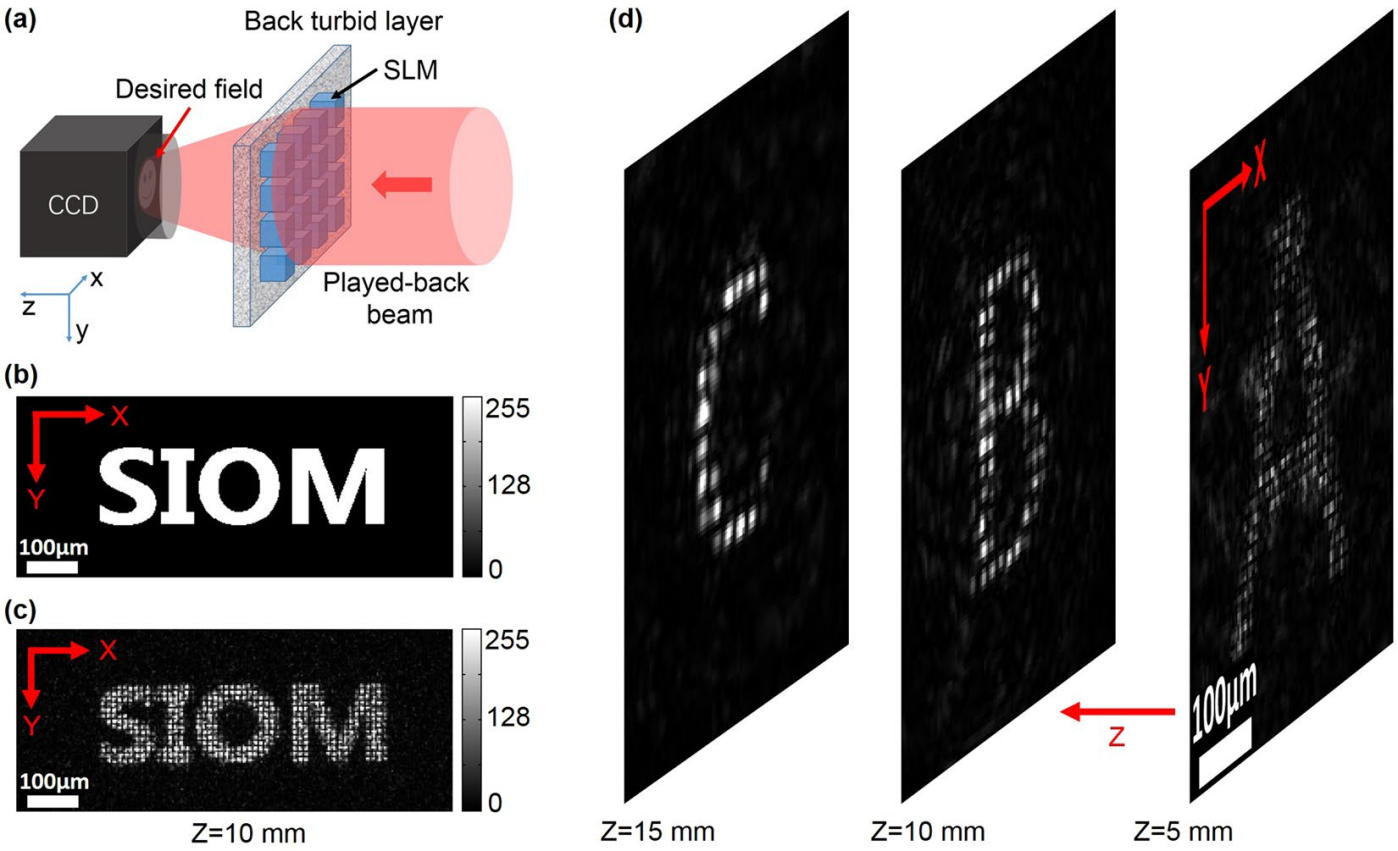
Controlling light between two ground glass plates. (**a**) Experimental setup for observation of the projected intensity patterns. (**b**) Intended 2D intensity pattern of capital letters ‘SIOM’ (0.55 mm * 0.15 mm) on an x-y plane 10 mm away from the back ground glass plate. (**c**) Image of the generated/projected 2D intensity pattern on the target x-y plane. The phase map *H* is calculated from the intensity pattern shown in (**b**) with an iterative Fourier transformation algorithm. (**d**) Images of the generated/projected 3D intensity patterns on three x-y planes with z-positions of 5 mm, 10 mm and 15 mm, respectively. The phase map *H* in this case is a superposition of three phase maps which are calculated from three intensity patterns, capital letter ‘A’ (0.22 mm * 0.26 mm), capital letter ‘B’ (0.12 mm * 0.19 mm) and capital letter ‘C’ (0.13 mm * 0.17 mm), respectively, with the iterative Fourier transformation algorithm.

Here we used the iterative Fourier transformation algorithm (an in-built feature in the application software of our SLM) to calculate the phase-only wavefront *H* from the intended intensity distributions/patterns, and used a CCD camera (see Fig. 4a) to observe the actual intensity patterns generated/projected by *H*. Figure 4b and Fig. 4c show the intended and observed intensity pattern, respectively. Similar to the multi-foci case, to project a 3D intensity distribution, the *H* was calculated by the following process. First, the iterative Fourier transformation algorithm was used to calculate three phase-only *H* s for three 2D intensity patterns, *i.e*., capital letter ‘A’, ‘B’ and ‘C’ with their target z positions at 5 mm, 10 mm and 15 mm, respectively. The second and third procedures were the same as the multi-foci case. Figure 4d shows the observed 3D intensity patterns on the CCD camera.

In this case, the distortions between the intended and the observed intensity patterns appear as speckle artefacts. Simulations showed that with an amplitude-phase SLM, smooth intensity distributions could be obtained (Supplementary Information Fig. S3).

## Discussion

Here we proposed and demonstrated a non-invasive approach for 3D light manipulation between two turbid layers. A direct application of our method is to focus light non-invasively in between turbid layers, when no fluorophores, second harmonic particles or ultrasound focuses exist, such as in eggs and crustaceans.

A previous memory effect based imaging method^44^ used an adaptive optimization method to find the optimized phase pattern for the SLM, which corrected the initially speckled point spread function of a point source behind a thin scattering medium into a bright spot on the camera. Then due to the memory effect, objects in the vicinity of the pre-corrected point source could be directly imaged onto the camera. This mechanism looks very like ours, however, the fundamental distinction is that this method images objects passively, but ours controls light actively.

Ryu *et al*. reported an approach^52^ that transformed a single scattering layer into a scattering lens through a DOPC system, aiming to achieve variable focusing and 3D patterning. In contrast, our approach uses a surface quasi-point source for pre-characterization of a scattering sample and thus achieves focusing and 3D control of light in the space confined by turbid layers. Besides, their approach needed to measure multiple wavefronts of a reference point at different axial locations. In contrast, our approach, by utilizing the large longitudinal memory effect range, just performs a single wavefront detection and then achieves 3D light control over an axial range (16 mm) that nearly covers the distance (20 mm) between the two turbid layers.

Low frame rate of our SLM (60 Hz) limits our approach to those applications in which the decorrelation time of the back turbid layer is no less than 16 ms, while this time for *in-vivo* tissues is typically under 10 ms^53,54^. However, we believe this limitation can be easily broken with a high-speed SLM^55,56^.

There is an interesting phenomenon about the resolution of our approach. Theoretically, if the SLM is infinitely large so that all the light emitted from the quasi-point source is recorded and then phase-conjugated, the time-reversed focus should have the same size as the quasi-point source. For example, if the size of the quasi-point source is 10 μm, then the ideal time-reversed focus should also be 10 μm. Surprisingly, however, with a SLM of finite size (2 mm * 2 mm area), a quasi-point source of 10 μm diameter, and a 20 mm distance between the two turbid layers, as shown in the simulation part of the article, the size of the time-reversed focus is of diffraction-limited value 5.3 μm, smaller than that of the ideal time-reversal. Here, we give a possible explanation for this phenomenon. The quasi-spherical wave emitted from a quasi-point source with finite size can be seen as a superposition of many displaced spherical waves emitted from different points inside the quasi-point source. Phase gradient in the paraxial region of a spherical wave is smaller than that in the abaxial region, so if the quasi-point source is small enough and the SLM is placed in the paraxial region, the quasi-spherical wave received by the SLM can be approximated as a spherical wave, therefore, the size of the time-reversed focus is determine by the aperture of the SLM and the distance between the focus and the SLM. In contrast, an infinitely large SLM receives all the wavefront emitted from the quasi-point source, including the paraxial part and the abaxial part, so the size of the time-reversed focus should be exactly the same as the quasi-point source. Partial phase conjugation is generally considered to be inferior to complete phase conjugation because of its disadvantages, such as background noise around the time-reversed focus and speckle artefacts in the holographic optical field (Fig. 4c,d). However, with proper geometries, including the size of the quasi-point source, the distance between the two turbid layers and the size of the SLM, partial phase conjugation has better resolution than complete phase conjugation. Nevertheless, as the size of the quasi-point source increases, the quasi-spherical wave will have more high-frequency components, resulting in more energy transferred from the central focus to the surrounding speckles on the focal plane, therefore decaying the resolution.

Due to the capability of 3D light control, our approach may find applications in photodynamic therapy and optogenetics. Besides, when combined with ghost imaging^57^ or compressed sensing^58^, it can achieve 3D imaging between turbid layers.

## Methods

A diagram of the complete experimental system is shown in Supplementary Information Fig. S1. We used a continuous laser with 50 m coherent length and 100 mW output power. A full-wave liquid crystal variable retarder (LCC1413-B, Thorlabs, USA) was used to change the optical path difference between the sample beam and the reference beam during the recording step. The diameter of the reference beam was 33 mm after expanded by a 10X beam expander (BE10–532, Thorlabs, USA), which was about five times larger than the controlled area of our SLM (a circle area with a diameter of 6.9 mm). Because of the Gaussian-profile of the reference beam, larger beam diameter results in more uniform intensity distributions in its illuminating area on the SLM, and thus makes fuller use of the dynamic range of the interferogram-recording CCD camera. The ratio of the minimum to the maximum intensities of the reference beam within the controlled area of the SLM was 0.91. We used previously reported methods^59,60^ to align the DOPC system, and correct the aberration in the reference beam and the substrate curvature of the SLM.

### Data Availability

The datasets generated and analyzed during the current study are available from the corresponding author on reasonable request.

## Electronic supplementary material

Supplementary Information
