# Author Correction: Non-invasive three-dimension control of light between turbid layers using a surface quasi-point light source for precorrection

**DOI:** 10.1038/s41598-018-23757-w

**Published:** 2018-04-12

**Authors:** Mu Qiao, Honglin Liu, Guanghui Pang, Shensheng Han

**Affiliations:** 1 Key Laboratory for Quantum Optics and Center for Cold Atom Physics, Shanghai Institute of Optics and Fine Mechanics, Chinese Academy of Sciences, Shanghai, 201800 China; 2 University of Chinese Academy of Sciences, Beijing, 100049 China

Correction to: *Scientific Reports* 10.1038/s41598-017-10450-7, published online 29 August 2017

The original version of this Article omitted an additional affiliation for the authors Mu Qiao, Honglin Liu and Shensheng Han. The correct affiliations for these authors are listed below:

Key Laboratory for Quantum Optics and Center for Cold Atom Physics, Shanghai Institute of Optics and Fine Mechanics, Chinese Academy of Sciences, Shanghai, 201800.

University of Chinese Academy of Sciences, Beijing, 100049, China.

This has now been corrected in the HTML and PDF versions of this Article.

