# Supplementary material for: Non-invasive three-dimension control of light between turbid layers using a surface quasi-point light source for precorrection
Source: Sci Rep. 2017 Aug 29;7:9792. doi: 10.1038/s41598-017-10450-7 (PMC5575110; doi:10.1038/s41598-017-10450-7)
Supplement: Supplementary file 1 — Supplementary Information [file 41598_2017_10450_MOESM1_ESM.pdf]

Non-invasive three-dimension control of light between turbid layers using a surface quasi-point light source for precorrection : Supplementary Information

Mu Qiao, Honglin Liu*, Guanghui Pang, and Shensheng Han

Key Laboratory for Quantum Optics and Center for Cold Atom Physics, Shanghai Institute of Optics and Fine Mechanics, Chinese Academy of Sciences, Shanghai 201800, China.

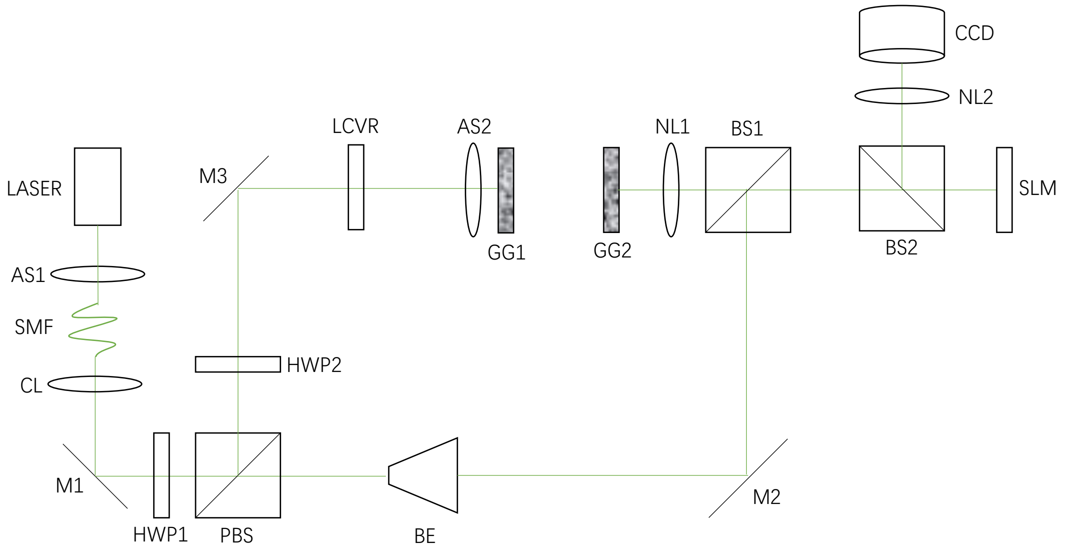

Figure S1. Experimental setup of our approach for 3D light control between two ground glass plates. Abbreviations: AS: aspheric lens, SMF: single mode fiber, CL: collimating lens, M: mirror, HWP: half wave plate, PBS: polarization beam splitter, BE: beam expander, LCVR: liquid crystal variable retarder, GG: ground glass plate, NL: Micro-Nikkor 105 mm f/2.8, Nikon, BS: beam splitter, SLM: spatial light modulator, CCD: CCD camera.

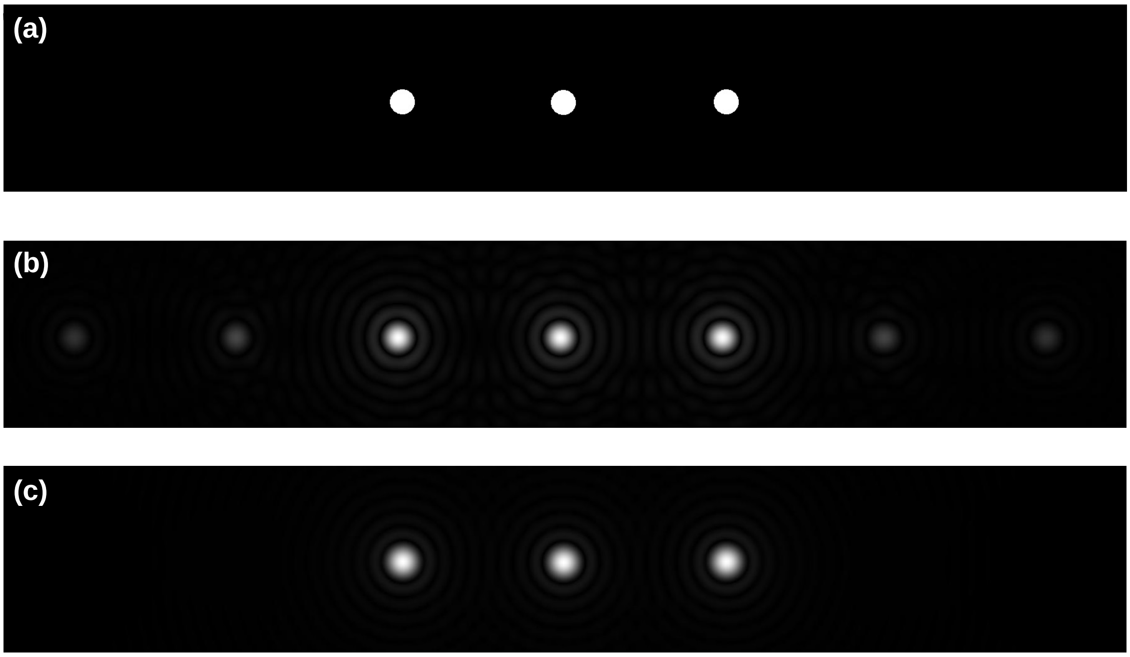

Figure S2. Simulation of the differences between multi-point focusing with phase-only wavefront and amplitude-and-phase wavefront. Parameters: the size of the quasi-point source on the internal surface of the front ground glass plate (right surface of GG1, Fig. S1) was 1 μm, the distance between the two ground glasses was 20 mm, the target x-y plane of the intended multi-foci was 10 mm away from the back ground glass plate (GG2, Fig. S1) and the SLM image on the external surface of the back ground glass plate (right surface of GG2, Fig. S1) was a circle area with a diameter of 2 mm. Here, we simplified GG2 as a random phase modulating layer with zero thickness. **(a)** Intended intensity pattern of multi-foci. The diameter of each focus was 2 μm, and the distance between two adjacent spots was 13 μm. **(b)** Projected/generated multi-foci by a phase-only wavefront, which was calculated from the pattern shown in (a) with an iterative Fourier transformation algorithm. The diameter of each focus was of diffraction-limited value 2.67 μm. In addition to the intended three foci on the center, high-order foci existed on both sides which was caused by the high-order harmonics in the phase-only wavefront. We contributed these high-order harmonics to the iterative Fourier transformation algorithm in which only phase information was conserved and the amplitude information was abandoned during the iteration. **(c)** Projected/generated multi-foci by an amplitude-and-phase wavefront. In this case, high-order foci disappeared (actually became very weak). The diameter of each focus was also of diffraction-limited value 2.67 μm.

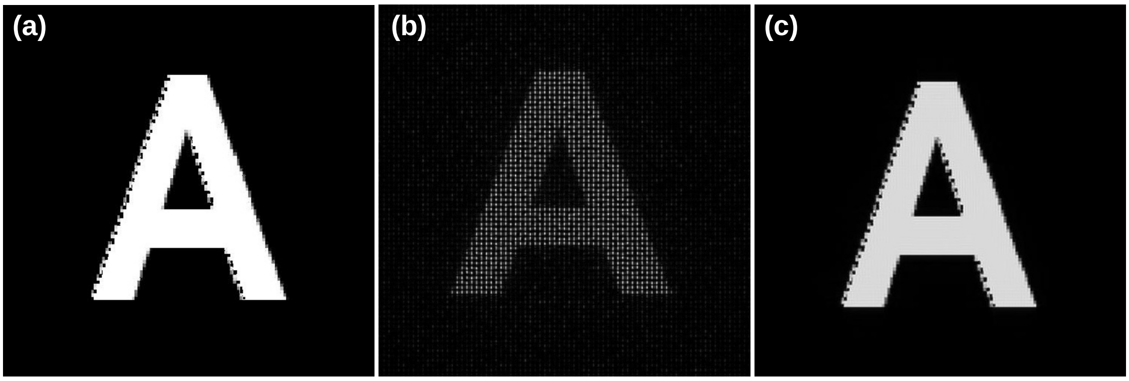

Figure S3. Simulation of the differences between intensity patterns projected by phase-only wavefront and amplitude-and-phase wavefront. Parameters were the same as the simulation shown in Fig. S2. **(a)** Intended intensity pattern of capital letter 'A' which was 0.27 mm in width and 0.31 mm in height. **(b)** Projected intensity pattern by a phase-only wavefront, which was calculated from the intended intensity pattern shown in (a) with an iterative Fourier transformation algorithm. Although the profile of the projected pattern was the same as the intended one, speckle artefacts existed in the projected pattern. **(c)** Projected intensity pattern by an amplitude-and-phase wavefront. In this case, speckle artefacts disappeared and the intensity distributions became smooth.
